# Spontaneous breathing promotes lung injury in an experimental model of alveolar collapse

**DOI:** 10.1038/s41598-022-16446-2

**Published:** 2022-07-25

**Authors:** María Consuelo Bachmann, Pablo Cruces, Franco Díaz, Vanessa Oviedo, Mariela Goich, José Fuenzalida, Luis Felipe Damiani, Roque Basoalto, Yorschua Jalil, David Carpio, Niki Hamidi Vadeghani, Rodrigo Cornejo, Maximiliano Rovegno, Guillermo Bugedo, Alejandro Bruhn, Jaime Retamal

**Affiliations:** 1 Departamento de Medicina Intensiva, Facultad de Medicina, Pontificia Universidad Católica de Chile, Santiago, Chile; 2 Escuela de Medicina Veterinaria, Facultad de Ciencias de la Vida, Universidad Andrés Bello, Santiago, Chile; 3 Unidad de Paciente Crítico Pediátrico, Hospital El Carmen de Maipú, Santiago, Chile; 4 Escuela de Postgrado, Universidad Finis Terrae, Santiago, Chile; 5 Departamento de Ciencias de La Salud, Carrera de Kinesiología, Pontificia Universidad Católica de Chile, Santiago, Chile; 6 Institute for Biological and Medical Engineering, Schools of Engineering, Medicine and Biological Sciences, Pontificia Universidad Católica de Chile, Santiago, Chile; 7 Unidad de Pacientes Críticos, Departamento de Medicina, Hospital Clínico Universidad de Chile, Santiago, Chile

**Keywords:** Translational research, Respiratory distress syndrome

## Abstract

Vigorous spontaneous breathing has emerged as a promotor of lung damage in acute lung injury, an entity known as “patient self-inflicted lung injury”. Mechanical ventilation may prevent this second injury by decreasing intrathoracic pressure swings and improving regional air distribution. Therefore, we aimed to determine the effects of spontaneous breathing during the early stage of acute respiratory failure on lung injury and determine whether early and late controlled mechanical ventilation may avoid or revert these harmful effects. A model of partial surfactant depletion and lung collapse was induced in eighteen intubated pigs of 32 ±4 kg. Then, animals were randomized to (1) SB‐group: spontaneous breathing with very low levels of pressure support for the whole experiment (eight hours), (2) Early MV-group: controlled mechanical ventilation for eight hours, or (3) Late MV-group: first half of the experiment on spontaneous breathing (four hours) and the second half on controlled mechanical ventilation (four hours). Respiratory, hemodynamic, and electric impedance tomography data were collected. After the protocol, animals were euthanized, and lungs were extracted for histologic tissue analysis and cytokines quantification. SB-group presented larger esophageal pressure swings, progressive hypoxemia, lung injury, and more dorsal and inhomogeneous ventilation compared to the early MV-group. In the late MV-group switch to controlled mechanical ventilation improved the lung inhomogeneity and esophageal pressure swings but failed to prevent hypoxemia and lung injury. In a lung collapse model, spontaneous breathing is associated to large esophageal pressure swings and lung inhomogeneity, resulting in progressive hypoxemia and lung injury. Mechanical ventilation prevents these mechanisms of patient self-inflicted lung injury if applied early, before spontaneous breathing occurs, but not when applied late.

## Introduction

Patients with severe lung injury usually have a high respiratory drive, resulting in an intense inspiratory effort^1^. The patient’s effort has emerged as a potential driver of damage to the lungs, coining the concept of “patient self-inflicted lung injury” (P-SILI)^2,3^. Nevertheless, this phenomenon is based only on clinical observations and its pathophysiological plausibility. The proposed mechanisms involved in P-SILI may be similar to those classically described for ventilator-induced lung injury (VILI), including uneven ventilation distribution associated with local and global lung strain exceeding the thresholds for lung damage^2,4,5^.

Harmful effects of spontaneous effort have been demonstrated in experimental models of uninjured and injured lungs under mechanical ventilation (MV), but mechanisms are still poorly understood^6,7^. Spontaneously breathing patients with acute hypoxemic respiratory failure (AHRF), who failed a trial of non-invasive ventilation, presented higher minute ventilation and markedly higher levels of intrathoracic pressure swings than patients that did not fail. The therapeutic implications of this concept are of utmost importance, as starting invasive MV in patients with AHRF could become a protective strategy by stopping the detrimental effects associated to high respiratory drive^8^.

We aimed to determine the effects of spontaneous breathing (SB) on lung injury and inflammation, as well as the associated mechanisms, during the initial phase of acute respiratory failure. In addition, we wanted to determine whether early or late controlled protective MV can modify these effects. We hypothesize that SB may further injure the lungs due to the presence of vigorous inspiratory efforts, generating a heterogeneous distribution of ventilation and increasing the risk of P-SILI. Early and late controlled MV can prevent or reverse these deleterious effects by decreasing intrathoracic pressure oscillations and improving regional air distribution.

## Methods

### Animal preparation

We studied 18 piglets (Sus scrofa domestica) of 2–3 months old, weighing 32 ± 4 kg. The animals were anesthetized with an intramuscular injection of xylazine (2 mg/kg) and ketamine (20 mg/kg), followed by a continuous intravenous infusion of ketamine (30 mg/kg/h), fentanyl (0.5–1.0 μg/kg/h), midazolam (0.1 mg/kg/h), and rocuronium (0.3 mg/kg/h). Ringer’s lactate 30 ml/kg/h was infused IV during the first hour. Then it was decreased to 10 ml/kg/h until the end of the experiment.

The animals were placed in supine position and, after tracheal intubation, were mechanically ventilated (Carescape R860, GE Healthcare, USA) in a volume-controlled ventilation (VCV) mode with the following settings: tidal volume (V_T_) of 8 ml/kg, respiratory rate (RR) 30 bpm, positive end-expiratory pressure (PEEP) 5 cmH_2_O, inspiratory to expiratory ratio (I:E) 1:2, and oxygen inspired fraction (F_I_O_2_) of 1. The animals were continuously monitored with electrocardiogram and pulse oximetry. Invasive systemic arterial pressure was monitored using the PICCO system (PV2015L20, Pulsion, Munich, Germany), placed in a femoral artery. A pulmonary artery catheter was inserted through the femoral vein under ultrasound viewing. A bladder catheter was surgically inserted to measure urine output per hour. NICO-monitor (Philips, Wallingford, CT, USA) was connected for volumetric capnography measurements.

### Experiment protocol

**Figure 1:**
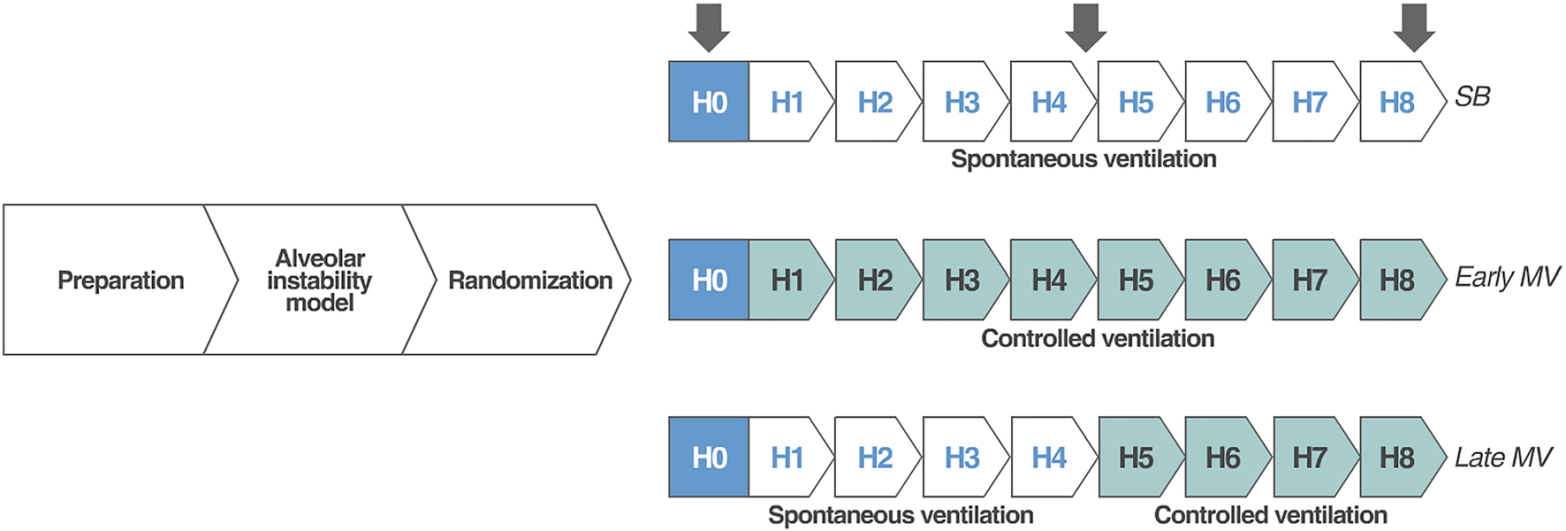
Study design and timeline. Preparation corresponds to anesthesia and invasive monitoring, which took around 2 h, baseline was measured at the end of this period. Alveolar collapse model corresponds to partial surfactant depletion and lung collapse model. After induction of the model, animals were randomized for spontaneous breathing, early controlled MV and late controlled MV by a period of 8 h. Arrows: data record points.

After the initial preparation, the pigs were stabilized for 30 min, and baseline measurements were recorded. We adapted a previously established model of alveolar instability based on partial surfactant depletion and lung collapse^9,10^. Briefly, deeply anesthetized animals on controlled MV received 30 ml/kg of warmed isotonic saline through the endotracheal tube. After 10 s, the saline solution was passively drained by gravity. Finally, endotracheal suction until SpO_2_ was less than 80% was performed. After an initial stabilization of 10 min, and before starting the 8-h-study period, animals were allocated into three groups by a six-sized block randomization using an online randomization tool (https://www.randomizer.org)^11^ (Fig. 1).

- SB: Animals were ventilated with pressure support ventilation (PSV) set, at the beginning, to achieve Vt of 6–8 ml/kg, PEEP between 0 and 5 cmH_2_O (to maintain SpO_2_ > 92%), and F_I_O_2_ of 1.
- Early controlled MV: Animals were ventilated with VCV using V_T_ of 6 to 8 ml/kg, RR of 30 breaths per minute (bpm), PEEP of 5 cmH_2_O, I:E ratio 1:2, and F_I_O_2_ of 1.
- Late controlled MV: Animals were ventilated with PSV with the same settings as the SB group for 4 h. Then they were switched to VCV (same settings as Early controlled MV group) for the next 4 h.

During controlled mechanical ventilation, pigs were anesthetized, keeping the initial infusion of ketamine, fentanyl, midazolam, and rocuronium. To switch to SB, we stopped midazolam and rocuronium infusion, keeping ketamine (30 mg/kg/h) and fentanyl (0.5–1.0 μg/kg/h) until the onset of ventilatory efforts sufficient to maintain VT close to 6 ml/kg. The respiratory effort was monitored clinically and looking at the inspiratory deflection of esophageal pressure. Animals were euthanized after completing the 8-h-study period.

### Measurements

Data were collected at (1) Baseline: before inducing the alveolar collapse model; (2) H0: after stabilization of alveolar collapse model (3) H4: 4 h after H0; (4) H8: 8 h after H0 at the end of the protocol (Fig. 1).

1. **Respiratory mechanics and gas exchange**
  Respiratory mechanics were assessed with a NICO monitor, using airway pressure and flow waveforms. For SB-group we considered Ppeak as the highest airway pressure during the respiratory cycle. Esophageal balloon-tipped catheters (NeuroVent Research Inc, Toronto, Canada) were placed, and pressures were monitored and registered through a double-channel pneumotachometer (Data Acquisition System, Hans Rudolf, Inc., USA). Gas exchange analysis was performed using a bedside blood analyzer (i-STAT®-1 immunoready).
2. **Electrical impedance tomography (EIT)**
  A 16 electrode-belt was placed in the mid-thoracic region, and continuous lung impedance was assessed by EIT (Pulmovista 500, Dräger Medical Systems, USA). Offline analysis of EIT data was performed, and the following parameters were calculated:
  Lung inhomogeneity was determined by the Global Inhomogeneity index (GI), which quantifies the homogeneity of the tidal volume distribution through quantitative lung pixel impedance dispersion^12^.
  Regional ventilation distribution was assessed by Impedance Ratio (IR), a parameter for determining the dependent and non-dependent regions' air distribution^13^. An IR > 1 represents a ventral distribution predominance, while an IR < 1 represents mainly a dorsal distribution.
  Tidal Variation of Impedance (TVI), representing impedance change generated by inspired gas during a respiratory cycle^14^.
  End Expiratory Lung Impedance (EELI) corresponds to the impedance value at the end of expiration^13,14^. Its changes have been correlated with changes in end-expiratory lung volume^15^.
  Regional Ventilation Delay (RVD), representing the temporal delay of lung regional ventilation, it is associated to ventilatory heterogeneity and lung tidal recruitment^16^.
3. Bronchoalveolar lavage
  Bronchoalveolar lavage (BAL) was performed with a fiberoptic bronchoscope (Olympus BF 3C40; Olympus Optical Co. Hamburg, Germany). Briefly, we instilled 20 ml of 0.9% saline solution at the ventral and dorsal regions of the left lung. Subsequently, we aspirated the fluid, and the recovered fluid was centrifuged, immediately frozen with liquid nitrogen, and stored at − 80ºC for posterior analysis^17^. This procedure was performed at H4 and H8.
4. Lung tissue analysis
  After euthanasia, during the opening of the chest wall, PEEP 5 cmH_2_O was set. Then, the inferior cava vein was sectioned, the trachea was clamped at end-expiratory lung volume, and the heart–lung piece was excised. Lung tissue samples were collected from dependent, non-dependent, and intermediate lung regions. Tissues were immersed in 10% buffered formalin, processed, and stained with hematoxylin–eosin for histological analysis^17,18^. Other samples from the same lung regions were immediately frozen in liquid nitrogen, kept at − 80 °C, and finally processed for biochemical and molecular biology analysis^18^.
5. Cytokine’s quantification
  By the Enzyme-Linked ImmunoSorbent Assay (ELISA) method, the concentrations of interleukin-8 (IL-8), tumor necrosis factor-alpha (TNF-α) and transforming growth factor-beta (TGF-β) as a pathological mechanotransduction-induced protein were measured in BAL fluid (BALF), and lung samples (tissue homogenates)^18^. The resulting concentrations in BALF were indexed by urea and in tissue by proteins^17^.
6. Histological score
  To assess lung damage, fixed and stained lung tissue samples were analyzed with light microscopy. A validated semiquantitative score was used to evaluate four parameters of lung injury: alveolar hemorrhage, alveolar and septal inflammation, and septal disruption; each of these categories received a score ranging from 0 to 4, where 0 corresponds to no pathologic alteration, 1 corresponds to mild, 2 corresponds to moderate, 3 corresponds to severe and 4 very severe pathologic alteration^19^. The average was reported of ten random areas for each section, at 200 × magnification^18^.
7. Statistical analysis

We calculated that a sample size of six animals per group (18 total) was needed to detect a difference of 3.5 ± 2 on the global histological score between groups, with a *p* < 0.05 and a power of 0.8. The Shapiro–Wilk test was used to test data for normality, and we expressed values as means—standard deviation (SD) or median—interquartile range, accordingly. Intragroup analysis was done using the Friedman test or one-way ANOVA, as appropriate, to study the effect of time over any variable (e.g., Late MV group). For comparisons over time between groups (Early MV and SB groups), regarding each physiological variable, we used a generalized linear mixed model (GLMM), adding a random effect by the subject (pig). Tukey pairwise multiple comparison test was used for posthoc comparisons for the effect of time within the group and between groups. The statistical analyses were conducted by RStudio 2022 (Integrated Development Environment, Boston, MA, USA) and by GraphPad Prism version 7.0 (GraphPad Software, San Diego, CA, USA). Statistical tests were carried out with the significance level set at *p* ≤ 0.05.

### Ethics approval and consent to participate

The study was approved by the Animal Ethics Committee in Pontificia Universidad Católica de Chile (ID 170315007) and Universidad Andrés Bello (ID 021/2018). The protocol was designed following the National Institute of Health’s guidelines (NIH) and reported in accordance with ARRIVE guidelines.

## Results

No significant differences were found between groups in body weight, hemodynamics, lung mechanics, and gas exchange at baseline (Table 1). The alveolar collapse model was well tolerated and induced an immediate effect on oxygenation and lung mechanics in all the animals.

**Table 1:** Baseline physiologic data for the experimental groups.

Variable	SB	Early MV	Late MV	*p* value
	Median (IQR)	Median (IQR)	Median (IQR)	
Weight (kg)	32 (29–36)	36 (27–36)	30 (26–33)	0.541
HR (bpm)	77 (55–89)	72 (66–84)	62 (45–93)	0.948
MAP (mmHg)	84 (73–90)	90 (83–92)	91 (80–101)	0.300
CO (l/min)	2.7 (2.5–3.8)	3.7 (3.2–4.3)	3.4 (3.0–3.8)	0.222
EVLW (ml)	351 (276–404)	318 (261–351)	322 (285–571)	0.383
ITBV (ml)	637 (590–722)	722 (611–850)	752 (698–849)	0.216
GEDV (ml)	510 (472–579)	569 (490–681)	639 (560–761)	0.137
Crs (ml/cmH_2_O)	30 (24–38)	28 (18–32)	34 (28–38)	0.321
PaO_2_/F_I_O_2_	442 (423–513)	441 (367–464)	371 (304–431)	0.071
PaCO_2,_ mmHg	43.5 (27–68)	39.6 (31–51)	43.8 (39–72)	0.172
pH	7.4 (7.2–7.5)	7.5 (7.4–7.6)	7.3 (7.1–7.4)	0.055

MV: mechanical ventilation, SB: spontaneous breathing, HR: heart rate, MAP: mean arterial pressure, CO: cardiac output, EVLW: extravascular lung water, ITBV: intrathoracic blood volume, GEDV: global end diastolic volume, Crs: respiratory system compliance.

### Effects of spontaneous ventilation

#### SB versus Early MV groups

**Figure 2:**
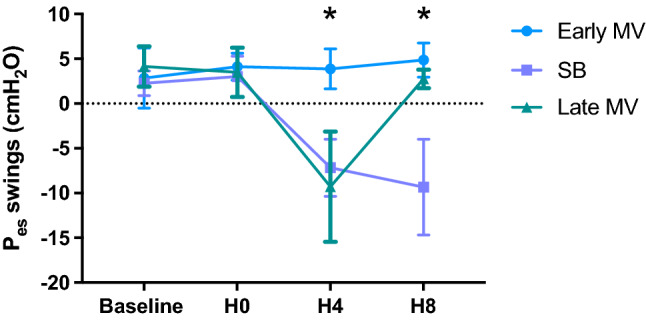
Changes in esophageal pressure swings over time in the three ventilatory groups. MV corresponds to mechanical ventilation, SB corresponds to spontaneous breathing, Pes corresponds to esophageal pressure. *p < 0.05.

**Figure 3:**
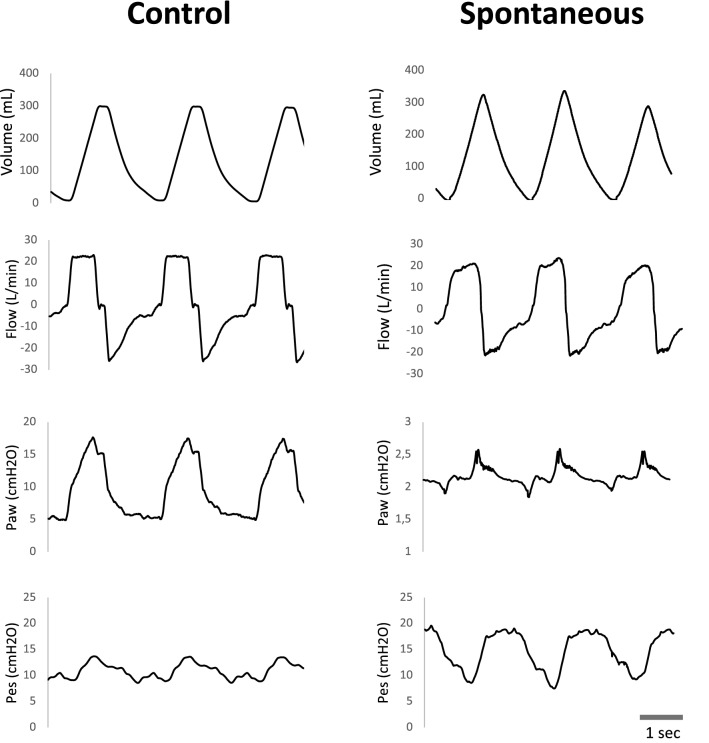
Representative tracing of Volume, Airflow (Flow), Airway pressure (Paw) and Esophageal pressure (Pes) from two animals from the Early MV and SB group. The scale of Paw in the SB animal was adjusted to improve visualization.

**Figure 4:**
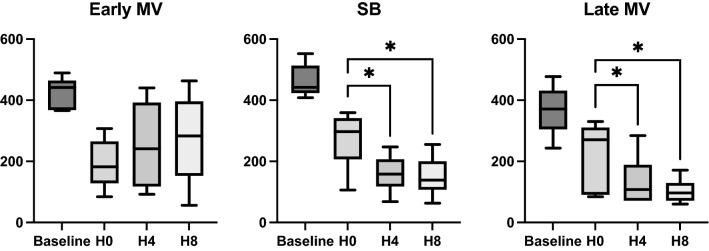
Changes in PaO_2_/FIO_2_ over time in SB-group, Early MV group, and Late MV group. *p < 0.05, using GLMM for the SB-group, and the Friedman test for the Late VM group.

**Figure 5:**
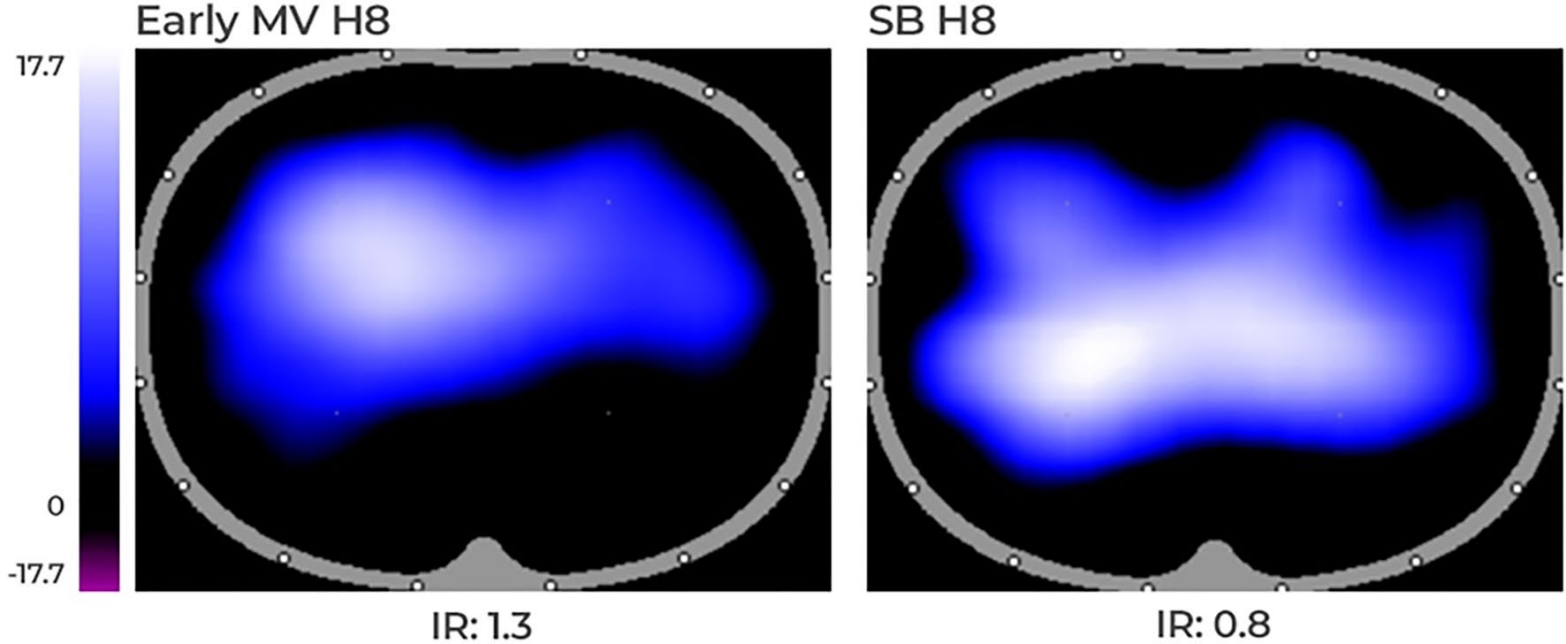
Representative images of an animal from the SB-group (right) and another from the Early MV group (left). The pulmonary ventilation distribution assessed by electric impedance tomography showed that SB presented dorsal predominant ventilation, as is shown by the impedance ratio = 0.8; in contrast, Early MV ventilation was predominantly ventral, as is shown by the impedance ratio = 1.3. *IR* impedance ratio.

**Figure 6:**
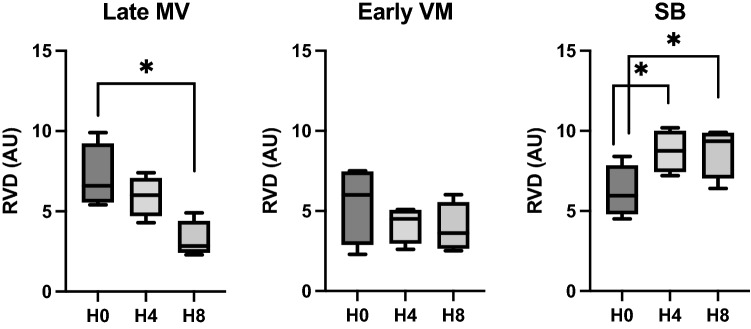
Regional ventilation delay values from SB and Early MV and Late MV groups. *MV* mechanical ventilation, *SB* spontaneous breathing, *RVD* regional ventilation delay, *AU* arbitrary units. *p < 0.05, using GLMM for the SB-group, and the Friedman test for the Late VM group.

**Figure 7:**
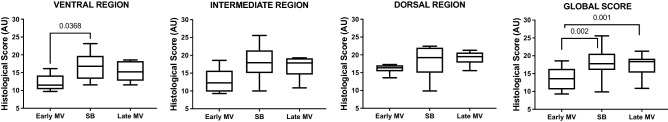
Regional and global histological lung injury score. *MV* mechanical ventilation, *SB* spontaneous breathing, *AU* arbitrary units. *p < 0.05.

- **Hemodynamics**
  No differences were found in hemodynamics over time. Cardiac output showed interaction, increasing from H0 to H4, and then decreasing at H8 in the Early MV group. (Table 2).
- **Respiratory mechanics and gas exchange**
  We observed interaction with time and a group effect in RR and PEEP. Changes in PEEP were expected due to the different settings defined by protocol. RR showed a progressive increase from H0 to H8 in the SB group and remained higher than the Early MV group at H4 and H8. (Table 3).
  Esophageal pressure swings showed progressive increment between H0 to H4, and H0 and H8 in SB group. Differences between groups were evident at H4 and H8. interaction between groups and time were positive (Figs. 2, 3).
  PaO_2_/F_I_O_2_ ratio also showed interaction (*p* = 0.003), but without time or group effect separately, if not as a whole. In the SB-group, we observed a progressive decrease in PaO_2_/F_I_O_2_ ratio over time (*p* = 0.02), in contrast to the Early MV group, which increased PaO2/FIO2 ratio above 200 mmHg and then remained stable over time. At the end of the experiment, the Early MV group appeared to have higher oxygenation, a difference which was on the border of statistical significance (138 [107–200] vs. 283 [153–396], *p* = 0.053), (Fig. 4).
- **Electrical impedance tomography**
  We observed a progressive dorsal ventilation distribution in the SB-group throughout the study period, as reflected by lower values in IR (Table 4, Fig. 5 and Figure Suppl 1). The early VM group showed no changes over time in the IR. Additionally, there was a progressive increase in temporal heterogeneity of ventilation in the SB-group, as expressed by the increase in RVD along time (Fig. 6).
- **Cytokines in bronchoalveolar lavage fluid, plasma, and lung tissue**
  When analyzing the concentration of pro-inflammatory cytokines in regional BALF, and lung tissue, in both groups, no significant differences were observed. (Figure Suppl 2 and 3).
- **Histological analysis**
  We observed a higher global histological score in the SB-group than in the Early MV (*p* = 0.002) (Fig. 6). Regionally, the main difference between these groups was found in the ventral region, while no differences were found in the intermediate and dorsal areas (Fig. 7 and Suppl 4).

**Table 2:** Hemodynamic parameters.

Variable	Group	Hour 0	Hour 4	Hour 8	*p value*	Group effect	Time effect	Interaction
		Median (IQ)	Median (IQ)	Median (IQ)				
HR (bpm)	SB	54 (39–75)	63 (43–96)	68 (46–89)		NS	0.006	NS
	Early MV	58 (45–65)	75 (63–112)	86 (63–124)				
	Late MV	62 (54–68)	72 (54–125)	122 (89–156)	NS			
MAP (mmHg)	SB	80 (79–97)	88 (82–105)	80 (71–105)		NS	NS	NS
	Early MV	98 (82–112)	106 (90–114)	103 (80–120)				
	Late MV	94 (84–115)	121 (99–158)	103 (90–136)	NS			
CO (l/min)	SB	2.9 (2.5–3.8)	3.1 (2.8–4.1)	3.5 (2.9–5.1)		NS	NS	NS
	Early MV	3.2 (2.4–4.9)	4.2 (3.6–5.2)	3.2 (2.5–3.8)				
	Late MV	3.2 (2.8–4.7)	4.5 (3.5–5.5)	4.2 (3.4–4.8)	NS			
EVLW (ml)	SB	540 (467–691)	537 (379–846)	609 (443–973)		NS	NS	NS
	Early MV	471 (381–544)	450 (408–598)	405 (318–645)				
	Late MV	688 (503–1254)	694 (508–1029)	506 (369–667)	NS			
ITBV (ml)	SB	806 (743–859)	810 (727–928)	742 (731–1192)		NS	NS	NS
	Early MV	674 (606–851)	869 (662–1036)	735 (629–846)				
	Late MV	840 (760–903)	1007 (818–1285)	790 (672–924)	NS			
GEDV (ml)	SB	645 (594–687)	648 (582–743)	594 (567–953)		NS	NS	NS
	Early MV	615 (485–680)	695 (530–828)	588 (503–677)				
	Late MV	672 (608–723)	805 (655–1028)	651 (538–786)	NS			

*p* value corresponds to Friedman test to the Late MV group, (*) correspond to *p* < 0.05 for multiple comparisons. The interaction, group effect, and time effect were estimated between SB and Early MV groups, using the GLMM regarding the combined effects of time and ventilatory strategy over each variable. The uppercases * and ◊, denote Tukey test for comparing variables over time in case of the interaction was present.

*MV* mechanical ventilation, *SB* spontaneous breathing, *HR* heart rate, *MAP* median arterial pressure, *CO* cardiac output, *EVLW* extravascular lung water, *ITBV* intrathoracic blood volume, *GEDV* global end diastolic volume.

**Table 3:** Ventilatory parameters.

Variable	Group	Hour 0	Hour 4	Hour 8	*p* value	Group effect	Time effect	Interaction
		Median (IQ)	Median (IQ)	Median (IQ)				
V_T_ (ml/kg)	SB	7.2 (5.5–7.9)	6.5 (5.8–7.3)	5.3 (4.7–6.5)		NS	NS	NS
	Early MV	6.5 (4.8–7.3)	6.5 (4.6–7.2)	6.1 (4.6–6.6)				
	Late MV	8.1 (7.4–9.0)	7.8 (6.8–9.6)	6.1 (5.5–6.8)	NS			
RR (bpm)	SB	30 (30–30)*◊	39 (31–43)+*	36 (33–42)¡◊		0.01	0.01	0.01
	Early MV	30 (30–30)	30 (30–30)+	30 (30–30)¡				
	Late MV	30 (30–30)	30 (26–45)	30 (30–30)	NS			
PEEP (cmH_2_O)	SB	5 (5–5)*◊	2 (1–3)+*	2 (1–3)¡◊		0.05	0.01	0.01
	Early MV	5 (5–5)	5 (5–5)+	5 (5–5)¡				
	Late MV	5 (5–5)*	2 (2–3)* ◊	5 (5–5)◊	0.001			
P_peak_ (cmH_2_O)	SB	22 (14–26)	6 (5–9)+	6 (4–8)¡		0.002	0.008	NS
	Early MV	23 (20–41)	22 (21–23)+	24 (18–27)¡				
	Late MV	21 (12–29)*	7 (5–11)*	21 (18–27)	0.006			
P_plateau_ (cmH_2_O)	SB	18 (16–21)	NA	NA		NS	NS	NA
	Early MV	20 (16–24)	20 (18–21)	23 (16–24)				
	Late MV	17 (16–19)	NA	17 (15–22)	NS			
P_mean_ (cmH_2_O)	SB	10 (9–12)	2 (0.7–3.5)+	2 (0.8–4.2)¡		0.001	0.02	NS
	Early MV	11 (8.7–15)	11 (8.7–11.3)+	11 (8.7–12)¡				
	Late MV	9 (5.8–11)*	2 (1.8–3.8)*◊	10.5 (8.8–13)◊	0.008			
DP (cmH_2_O)	SB	15 (11.8–17)	NA	NA		NS	NA	NA
	Early MV	15 (11.8–27)	15 (13–16)	17 (11.2–19-2)				
	Late MV	12 (11–14)	NA	11.5 (7.3–14)	NS			
C_RS_ (ml/cmH_2_O)	SB	15.6 (13–20)	NA	NA		NS	NS	NA
	Early MV	13.5 (8.6–18.6)	14 (9.9–19)	11 (8.6–20.8)				
	Late MV	21.5 (15.5–23)	NA	16.6 (8.5–20)	NS			
V_min_ (l/min)	SB	7.8 (6.3–9)	6.9 (6–8.3)	6.4 (5.5–8)		NS	NS	NS
	Early MV	6.5 (3.1–7.5)	6.5 (4.5–7)	6 (4.6–6.7)				
	Late MV	6.8 (5.5–7.6)	7.7 (6.5–8.7)	6 (5.4–6.5)	NS			

*p* value corresponds to Friedman test to the Late MV group, (* and ◊) correspond to *p* < 0.05 for Dunn`s multiple comparisons test. The interaction, group effect, and time effect were estimated between SB and Early MV groups, using the GLMM for each variable. Posthoc analysis was done using the Tukey test. The uppercases * and ◊, denote *p* < 0.05 in pairwise comparisons for time within each group. The “+” and “¡” signs denote *p* < 0.05 in pairwise comparisons between groups.

MV: mechanical ventilation, SB: spontaneous breathing, VT: tidal volume, RR: respiratory rate, PEEP: positive end-expiratory pressure, Ppeak: peak pressure, Pplateau: plateau pressure, Pmean: mean pressure, DP: driving pressure, CRS: respiratory system compliance, Vmin: minute volume.

**Table 4:** EIT parameters.

Variable	Group	Hour 0	Hour 4	Hour 8	*p* value	Group effect	Time effect	Interaction
		Median (IQ)	Median (IQ)	Median (IQ)				
GI	SB	0.99 (0.82–1.6)	1.14 (1.09–1.22)	1.22 (1.06–1.4)		NS	NS	NS
	Early MV	1.05 (1–1.17)	0.98 (0.96–1.01)	0.99 (0.97–1.04)				
	Late MV	1.02 (1–1.04)*	1.1 (1.1–1.4)*◊	1.04 (1–1.13)◊	0.006			
IR	SB	1.5 (1.2–2.7)	1.5 (0.8–1.9)	0.8 (0.5–1.1)		NS	0.002	0.04
	Early MV	1.6 (1.3–2.1)	1.5 (1.1–2)	1.4 (0.8–1.9)				
	Late MV	1.5 (1–2.2)*	0.8 (0.3–1.3)*	1.3 (0.5–2)	0.002			
TVI	SB	2460 (1455–4110)	1665 (1503–1944)	1959 (1431–3050)		NS	NS	NS
	Early MV	2393 (1456–3249)	2669 (2205–2892)	2429 (2157–2726)				
	Late MV	2558 (1909–3245)*	2227 (1581–2933)	1137 (876–1768)*	0.002			
EELI	SB	308 (156- 924)	187 (114–357)	93(54–130)		NS	NS	NS
	Early MV	169 (137–826)	254 (78–699)	205 (42–302)				
	Late MV	403 (77–1070)	373 (161–573)	229 (46–413)	NS			
RVD	SB	5.6 (4.8–7.9)*◊	8.7 (7.4–10)+*	9.3 (7–9.9)¡◊		0.009	NS	0.01
	Early MV	6 (2.9–7.5)	4.5 (2.9–5.1)+	3.6 (2.6–5.5)¡				
	Late MV	6.6 (5.5–9.2)*	6 (4.7–7)	2.9 (2.4–4.4)*	0.04			

*p* value corresponds to Friedman test to the Late MV group, (* and ◊) correspond to *p* < 0.05 for multiple comparisons. The interaction, group effect, and time effect were estimated between SB and Early MV groups, using the GLMM for each variable. Posthoc analysis was done using the Tukey test. The uppercases * and ◊, denote *p* < 0.05 in pairwise comparisons time within each group. The “+” and “¡” signs denote *p* < 0.05 in pairwise comparisons between groups.

GI: global inhomogeneity index, IR: impedance ratio, TVI: tidal variation of impedance, EELI: end-expiratory lung impedance, RVD: regional ventilation delay.

### Effects of switching to protective controlled MV after a spontaneous breathing period

#### Late MV group

**Figure 8:**
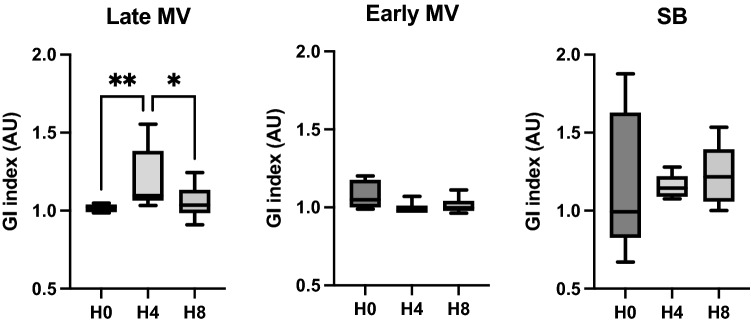
Global inhomogeneity index values from SB and Early MV and Late MV groups. *MV* mechanical ventilation, *SB* spontaneous breathing, *RVD* regional ventilation delay, *AU* arbitrary units. *p < 0.05, using the Friedman test for the Late VM group.

- **Hemodynamics**
  No differences were found in hemodynamics over time.
- **Respiratory mechanics and gas exchange**
  At the end of the SB period (H4), the presence of large esophageal pressure swings was observed (Fig. 2). Also, a significant decrease in PaO_2_/F_I_O_2_ was found at H4 which persisted after switching to protective MV (H8) (Fig. 4). No significant differences were observed in the other parameters examined.
- **Electrical impedance tomography**
  The Late MV group exhibited an increase in regional inhomogeneity (GI) during the spontaneous breathing period (1.09 [1.1–1.4] at H4 vs. 1.02 [0.9–1.03] at H0, *p* = 0.02); these changes were reversed during the protective MV period (1.03 [1–1.13] at H8, *p* = 0.02 vs H4) (Fig. 8). In addition, we observed a significant decrease in tidal ventilation (TVI at H0: 2558 [1909–3245] vs. H8: 1137 [876–1768], *p* = 0.01) and in temporal heterogeneity (RVD at H0: 6.6 [4.7–9.2] to H8: 2.9 [2.4–4.4], *p* = 0.04) (Table 4).
- **Cytokines in BALF, plasma, and lung tissue**
  No significant findings were observed when analyzing the concentration of pro-inflammatory cytokines in regional BALF or plasma. For lung tissue analysis, we did not find global or regional differences between group. (Figure Suppl 2 and 3).
- **Histological analysis**
  We observed that the Late MV group had a higher global histological score than the Early MV group, but it was comparable to that of the SB group. (Fig. 7 and Suppl 4).

## Discussion

The main findings of the present experimental study are, in the early phase of ARDS, animals breathing spontaneously had large esophageal pressure swings, predominantly dorsal ventilation, and an uneven temporal and spatial distribution of ventilation. This was associated with a more profound hypoxemia and higher lung injury after 8 h, compared to animals mechanically ventilated and without spontaneous breathing. Late connection to protective MV after 4 h of SB favored a more homogeneous ventilation; however, it failed to reverse hypoxemia or to prevent histological damage.

This study compares the effects of early and late connection to MV to prevent P-SILI on the initial phase of acute respiratory failure. Our group previously showed that SB (using very low tidal volume) presented higher dorsal ventilation than near-apneic controlled ventilation, but we did not observe differences in lung histological damage^19^. Our current experimental approach was slightly modified from previously published experiences^9,10^. We induced a model of lung instability and alveolar collapse, trying to avoid excessive inflammation from the model per se, which could mask the inflammation secondary to the ventilatory strategies studied. In addition, we included the Late MV group to extrapolate the condition of a patient with AHRF in whom intubation and connection to controlled MV are delayed. Thus, we tried to determine if controlled MV could reverse the potentially harmful effects of a previous period of spontaneous breathing with increased efforts. Contrary to some previous studies which examined SB as a trigger of lung injury during assisted MV^7,20^, we have examined the impact of SB with virtually no ventilatory support. Very low levels of support were used to prevent hypoventilation (average 1–2 cmH_2_O).

The putative underlying mechanisms for P-SILI are similar to those for VILI, and the majority of the evidence available for this concept derives from studies with rather low breathing efforts in the context of assisted MV^7,20^. However, during the COVID-19 pandemic, the frequent and controversial use of non-invasive MV, high flow nasal oxygenation and awake prone positioning to avert intubation, increased the interest in the risk of P-SILI during the SB period, particularly in those patients who were finally connected to invasive MV^5,21^. The first evidence supporting this hypothesis was reported by Mascheroni et al. several years ago. They showed that healthy sheep developed lung injury after inducing central hyperventilation. The animals presented progressive lung edema and hypoxemia during the protocol, which lasted 8.4 h on average^22^, a similar time frame as our study. However, the rest of the evidence that is usually cited to support this hypothesis is not derived from studies with spontaneous breathing and therefore its extrapolation to the context of the non-intubated patient with AHRF is highly debatable.

In this research, we observed that the Early MV group presented a progressive improvement in the PaO_2_/F_I_O_2_ ratio after the induction of the alveolar collapse model. In contrast, the SB group showed progressive deterioration in oxygenation over time (Fig. 4). This difference could be attributed to the use of a different PEEP setting between both groups^23^. However, we think the difference should be attributed to the whole strategy used (i.e., sedation, neuromuscular blocking, mechanical ventilation settings, among other factors). Although this deterioration in oxygenation may have reflected the progression of lung injury due to P-SILI, we can´t rule out that the lower PEEP used in the SB may have contributed to this result.

Tidal volume was similar in spontaneous and controlled ventilation; however, the higher and progressive intensity of intrathoracic pressure swings values observed in the SB group may suggest an increase in lung elastance or FRC diminishing, which could have mediated an increment in the volumetric strain of the remaining aerated lung tissue^22,24^. Regrettably, we did not measure lung elastance during SB to confirm these assumptions. In a similar model, Vimláti et al.^9,10^, demonstrated that this could be done using neuromuscular blockers intermittently. However, we expected that in this model the temporary muscular blocking could induce changes in the air distribution and lung collapse and then progressive reopening with variable temporal dynamics, which could induce a bias in the model. We tried to keep the model closer than we could to the clinical scenario.

We did not observe differences between groups over time regarding EELI. However, the analysis of the evolution of EELI in the SB group showed a progressive decrease throughout the study period. We can hypothesize that the SB-group needed higher inspiratory pressures to keep lung ventilation and to reopen collapsed lung units (tidal recruitment)^25–27^; this mechanism may be coupled to cyclic overdistension as well^28^.

Vascular stress secondary to high pulmonary blood flow^29^ and oscillations in the right ventricle stroke volume have been proposed to explain lung injury development during spontaneous breathing in different scenarios as during the practice of high-performance sports, and after upper airway obstruction in healthy humans^30^. Katira et al., showed that MV with intermittent high positive pressures induced marked oscillations in pulmonary blood flow. They suggested that it may be associated with microvascular injury and increased capillary leak due to capillary stress failure^31–34^. Blood flow oscillations could be intensified during respiratory failure and spontaneous breathing since the cyclic inspiratory negative pressures increase venous return, but without the resistance to vascular flow induced by PEEP (i.e., during CMV). We did not assess right ventricle dynamics during the protocol; however, we think this issue should be addressed in future research^35^.

EIT data showed that, unlike SB, during controlled MV, ventilation was distributed mainly to the non-dependent regions of the lungs. This can be explained due to the presence of alveolar collapse in the dependent areas, secondary to diaphragmatic paralysis (neuromuscular blockade), and low levels of PEEP used. Pellegrini and cols., showed in a porcine model of ARDS that the fraction of aerated lung was significantly smaller (baby lung) in controlled ventilation than during SB because, in the latter, the expiratory tonic activity of the diaphragm seems to preserve lung volume and protect against lung collapse^36^. Similarly, Yoshida and cols., evaluated the aeration changes with EIT and computed tomography (CT) during spontaneous efforts in mechanically ventilated patients with ARDS. They showed that the presence of SB efforts was associated with the movement of air from non-dependent to dependent zones (pendelluft phenomenon), causing tidal recruitment of dependent regions by concomitant collapse during expiration^6^. Therefore, beneficial, and harmful scenarios have been proposed depending on the intensity of the effort. The beneficial situation would be when mild spontaneous efforts keep the alveoli of the dependent pulmonary regions recruited^27^. On the contrary, a harmful situation would occur when vigorous regional negative pressures are associated with the pendelluft mechanism, the opening and closing of unstable lung units (atelectrauma), and more extensive heterogeneity in the distribution of ventilation, increasing the risk of VILI due to excessive lung strain^7,37^. We did not estimate pendelluft, however, we found important changes in the Regional Ventilation Delay index, suggesting the presence of repetitive recruitment-derecruitment phenomenon in the dorsal lung regions during spontaneous ventilation, denoting regional and temporal heterogeneity in lung ventilation^16^. Similarly, GI estimates the impedance pixel heterogeneity during tidal ventilation, which is correlated with the degree of pixel ventilation, comparing each individual pixel with the mean of all pixels from the lung field^12^. Both, RVD and GI point to greater heterogeneity in ventilation distribution during SB. In clinical research, heterogeneity is recognized as a marker of severity and mortality during ARDS^38^.

Recently, Hurtado and cols. found that spontaneous breathing in lung injury promotes regional strain and strain heterogeneity progression (P-SILI) in a murine model of alveolar instability. In contrast, protective MV prevented regional strain and heterogeneity progression in injured lungs. They conclude that the strain is associated with the diaphragm's vigorous contraction, resulting in an imperfect elastic anisotropic inflation (i.e., heterogeneity) and amplifying the regional lung injury^39^.

Regarding microstructural and inflammatory consequences of the ventilatory strategies studied, we found higher histological damage scores in SB and Late MV groups than early controlled MV. Surprisingly, we did not find greater histological damage in the dorsal regions in SB. Recently, in an ARDS animal model, Morais and cols. showed that assisted/controlled ventilation plus spontaneous efforts were associated with a higher histological score in the dependent lung regions^20^. Previously, Yoshida and cols. showed similar results^40^. However, these models were developed using a two-hit lung injury model with repeated lung lavages plus a variable period of injurious MV. A significant degree of inflammation could have been present since the beginning of the protocol due to the model itself^41^. Another remarkable difference is that we used PSV similar to clinical practice (very low levels), while in previous experiments, authors used assisted/controlled ventilation (PCV or APRV), allowing spontaneous efforts^6,7,20^.

Despite the observed differences in ventilatory distribution pattern, we did not find differences in the concentration of inflammatory mediators between SB and Early MV groups. Several reasons could explain this observation, such as a rather short time frame to detect changes in protein tissue concentrations, carry-over effect in the Late MV group, blood and lymphatic flow that could have opposite behavior than the ventilatory pattern, intrabronchial dissemination of inflammatory mediators, among others^42,43^.

Our study has several limitations. First, we used a model of lung collapse and surfactant depletion. This model alters lung mechanics and gas exchange but does not induce a formal lung injury and is not comparable to human ARDS. In pilot experiments we evaluated more severe models leading to lung injury but animals did not tolerate spontaneous ventilation. Second, the number of animals/group was rather low; some important parameters studied such as EIT indexes and cytokine levels presented high variability so the study may have been underpowered to find differences in some variables. Third, we observed by chance a lower severity of the alveolar collapse model at the beginning of the protocol in the Late MV group compared with the SB and Early MV groups. However, we think this factor did not influence our analysis, as most comparisons were performed between the SB and the Early MV group, while the Late MV group was analyzed mainly over time compared to itself. Fourth, In the Late MV group, some of the results observed at H8 may be explained by a carry-over effect. In this regard, some of the effects of the initial 4-h of spontaneous breathing could have influenced the results of H8. For example, inflammation or edema clearance/production are dynamic phenomena. Therefore, even though SB stopped, the dynamics of the process (i.e., inflammation or edema production) could continue, influencing the results of the next stage (H8). Fifth, we did not measure lung elastance during spontaneous breathing strategy, measuring of lung mechanics would have required to induce transient neuromuscular blocking, and we expected that it could induce changes in the air distribution and lung collapse, which could affect the interpretation of our conclusions*.*

## Conclusions

In conclusion, in this porcine model of acute lung collapse, we showed that marked inspiratory swing pressures associated to vigorous spontaneous breathing efforts induce heterogeneity in the distribution of ventilation, favoring the progression of lung injury. This observation supports the theoretical background that has been proposed to explain the P-SILI concept. The change to controlled MV, despite attenuating lung dysfunction, did not prevent lung tissue injury. Other mechanisms such as the presence of tidal recruitment and increased vascular and alveolar stress are yet to be studied.

## Supplementary Information

Supplementary Figure 1.

Supplementary Figure 2.

Supplementary Figure 3.

Supplementary Figure 4.

## Data Availability

Authors agree to make the data available. Correspondence and requests for materials should be addressed to JR (jaimeretamal@gmail.com).

## Abbreviations

SB: Spontaneous breathing
MV: Mechanical ventilation
P-SILI: Patient self-inflicted lung injury
VILI: Ventilator-induced lung injury
AHRF: Acute hypoxemic respiratory failure
VCV: Volume-controlled ventilation
VT: Tidal volume
RR: Respiratory rate
Bpm: Breaths per minute
PEEP: Positive end-expiratory pressure
I:E: Inspiratory to expiratory ratio
F_I_O_2_: Oxygen inspired fraction
SpO_2_: Pulse oximetry
PSV: Pressure support ventilation
PEEP: Positive end-expiratory pressure
GI: Global Inhomogeneity index
IR: Impedance ratio
TVI: Tidal variation of impedance
EELI: End expiratory lung impedance
RVD: Regional ventilation delay
BAL: Bronchoalveolar lavage
ELISA: Enzyme-linked immunosorbent assay
IL-8: Interleukin-8
TNF-α: Tumor necrosis factor-alpha
TGF-β: Transforming growth factor-beta
BALF: BAL fluid
SD: Standard deviation
ANOVA: Analysis of variance
PaO_2_/F_I_O_2_: Ratio of arterial oxygen partial pressure to fractional inspired oxygen
COVID-19: Coronavirus disease 2019
HR: Heart rate
MAP: Mean arterial pressure
CO: Cardiac output
EVLW: Extravascular lung water
ITBV: Intrathoracic blood volume
GEDV: Global end diastolic volume
CRS: Respiratory system compliance
Ppeak: Peak pressure
Pplateau: Plateau pressure
Pmean: Mean pressure
DP: Driving pressure
Crs: Respiratory system compliance
Vmin: Minute volume
Pes: Esophageal pressure
AU: Arbitrary units

## References

[1] Vaporidi K (2020). Respiratory drive in critically Ill patients pathophysiology and clinical implications. Am. J. Respir. Crit. Care Med..

[2] Brochard L, Slutsky A, Pesenti A (2017). Mechanical ventilation to minimize progression of lung injury in acute respiratory failure. Am. J. Respir. Crit. Care Med..

[3] Cruces P (2020). A physiological approach to understand the role of respiratory effort in the progression of lung injury in SARS-CoV-2 infection. Crit. Care.

[4] Yoshida T, Grieco DL, Brochard L, Fujino Y (2020). Patient self-inflicted lung injury and positive end-expiratory pressure for safe spontaneous breathing. Curr. Opin. Crit. Care.

[5] Grieco DL, Menga LS, Eleuteri D, Antonelli M (2019). Patient self-inflicted lung injury: Implications for acute hypoxemic respiratory failure and ARDS patients on non-invasive support. Minerva Anestesiol..

[6] Yoshida T (2013). Spontaneous effort causes occult pendelluft during mechanical ventilation. Am. J. Respir. Crit. Care Med..

[7] Yoshida T, Uchiyama A, Matsuura N, Mashimo T, Fujino Y (2012). Spontaneous breathing during lung-protective ventilation in an experimental acute lung injury model: High transpulmonary pressure associated with strong spontaneous breathing effort may worsen lung injury. Crit. Care Med..

[8] Carteaux G (2016). Failure of noninvasive ventilation for de novo acute hypoxemic respiratory failure: Role of tidal volume. Crit. Care Med..

[9] Vimláti L, Larsson A, Hedenstierna G, Lichtwarck-Aschoff M (2012). Haemodynamic stability and pulmonary shunt during spontaneous breathing and mechanical ventilation in porcine lung collapse. Acta Anaesthesiol. Scand..

[10] Vimláti L, Larsson A, Hedenstierna G, Lichtwarck-Aschoff M (2013). Pulmonary shunt is independent of decrease in cardiac output during unsupported spontaneous breathing in the pig. Anesthesiology.

[11] Haitsma JJ (2008). Ventilator-induced coagulopathy in experimental *Streptococcus pneumoniae* pneumonia. Eur. Respir. J..

[12] Zhao Z, Steinmann D, Guttmann J (2009). Global and local inhomogeneity indices of lung ventilation based on electrical impedance tomography. IFMBE Proc..

[13] Bachmann M (2018). Electrical impedance tomography in acute respiratory distress syndrome. Crit. Care.

[14] Frerichs I (2002). Detection of local lung air content by electrical impedance tomography compared with electron beam CT. J. Appl. Physiol..

[15] Hinz J (2003). End-expiratory lung impedance change enables bedside monitoring of end-expiratory lung volume change. Intensive Care Med..

[16] Muders T (2012). Tidal recruitment assessed by electrical impedance tomography and computed tomography in a porcine model of lung injury*. Crit. Care Med..

[17] Petersen AG (2021). Treatment with senicapoc in a porcine model of acute respiratory distress syndrome. Intensive Care Med. Exp..

[18] Retamal J (2016). High respiratory rate is associated with early reduction of lung edema clearance in an experimental model of ARDS. Acta Anaesthesiol. Scand..

[19] Araos J (2018). Near-apneic ventilation decreases lung injury and fibroproliferation in an near-apneic ventilation decreases lung injury and fibroproliferation in an acute respiratory distress syndrome model with extracorporeal membrane oxygenation. Am. J. Respir. Crit. Care Med..

[20] Morais CCA (2018). High positive end-expiratory pressure renders spontaneous effort noninjurious. Am. J. Respir. Crit. Care Med..

[21] Schmidt M (2021). Benefits and risks of noninvasive oxygenation strategy in COVID-19: A multicenter, prospective cohort study (COVID-ICU) in 137 hospitals. Crit. Care.

[22] Mascheroni D (1988). Acute respiratory failure following pharmacologically induced hyperventilation: An experimental animal study. Intensive Care Med..

[23] Spieth PM (2011). Open lung approach vs acute respiratory distress syndrome network ventilation in experimental acute lung injury. Br. J. Anaesth..

[24] Mauri T (2015). Effects of sigh on regional lung strain and ventilation heterogeneity in acute respiratory failure patients undergoing assisted mechanical ventilation*. Crit. Care Med..

[25] Pham T, Telias I, Beitler JR (2020). Esophageal manometry. Respir. Care.

[26] Wrigge H (2003). Spontaneous breathing improves lung aeration in oleic acid-induced lung injury. Anesthesiology.

[27] Wrigge H (2005). Spontaneous breathing with airway pressure release ventilation favors ventilation in dependent lung regions and counters cyclic alveolar collapse in oleic-acid-induced lung injury: A randomized controlled computed tomography trial. Crit. Care.

[28] Retamal J (2013). Preliminary study of ventilation with 4 ml/kg tidal volume in acute respiratory distress syndrome: Feasibility and effects on cyclic recruitment—derecruitment and hyperinflation. Crit. Care.

[29] Broccard AF (1998). Consequences of vascular flow on lung injury induced by mechanical ventilation. Am. J. Respir. Crit. Care Med..

[30] West JB (2006). Vulnerability of pulmonary capillaries during severe exercise. Br. J. Sports Med..

[31] Katira BH (2017). Adverse heart–lung interactions in ventilator-induced lung injury. Am. J. Respir. Crit. Care Med..

[32] Katira BH, Kuebler WM, Kavanagh BP (2018). Inspiratory preload obliteration may injure lungs via cyclical ‘on–off’ vascular flow. Intensive Care Med..

[33] West JB, Tsukimoto K, Mathieu-Costello O, Prediletto R (1991). Stress failure in pulmonary capillaries. J. Appl. Physiol..

[34] West JB, Mathieu-Costello O (1992). Stress failure of pulmonary capillaries: Role in lung and heart disease. Lancet.

[35] Vieillard-Baron A, Dreyfuss D (2017). Ventilator-induced lung injury: Follow the right direction! Another piece of the puzzle in the ventilator-induced lung injury epic. Am. J. Respir. Crit. Care Med..

[36] Pellegrini M (2017). The diaphragm acts as a brake during expiration to prevent lung collapse. Am. J. Respir. Crit. Care Med..

[37] Yoshida T, Uchiyama A, Matsuura N, Mashimo T, Fujino Y (2013). The comparison of spontaneous breathing and muscle paralysis in two different severities of experimental lung injury. Crit. Care Med..

[38] Cressoni M (2014). Lung inhomogeneity in patients with acute respiratory distress syndrome. Am. J. Respir. Crit. Care Med..

[39] Hurtado DE (2020). Progression of regional lung strain and heterogeneity in lung injury: Assessing the evolution under spontaneous breathing and mechanical ventilation. Ann. Intensive Care.

[40] Yoshida T (2017). Volume-controlled ventilation does not prevent injurious inflation during spontaneous effort. Am. J. Respir. Crit. Care Med..

[41] Borges JB (2015). Lung inflammation persists after 27 hours of protective Acute Respiratory Distress Syndrome Network Strategy and is concentrated in the nondependent lung. Crit. Care Med..

[42] Hedenstierna G, Lattuada M (2008). Lymphatics and lymph in acute lung injury. Curr. Opin. Crit. Care.

[43] Graf J (2009). Semi-quantitative tracking of intra-airway fluids by computed tomography. Clin. Physiol. Funct. Imaging.

