# Supplementary material for: Spontaneous breathing promotes lung injury in an experimental model of alveolar collapse
Source: Sci Rep. 2022 Jul 25;12:12648. doi: 10.1038/s41598-022-16446-2 (PMC9310356; doi:10.1038/s41598-022-16446-2)
Supplement: Supplementary file 1 — Supplementary Figure 1. [file 41598_2022_16446_MOESM1_ESM.pdf]

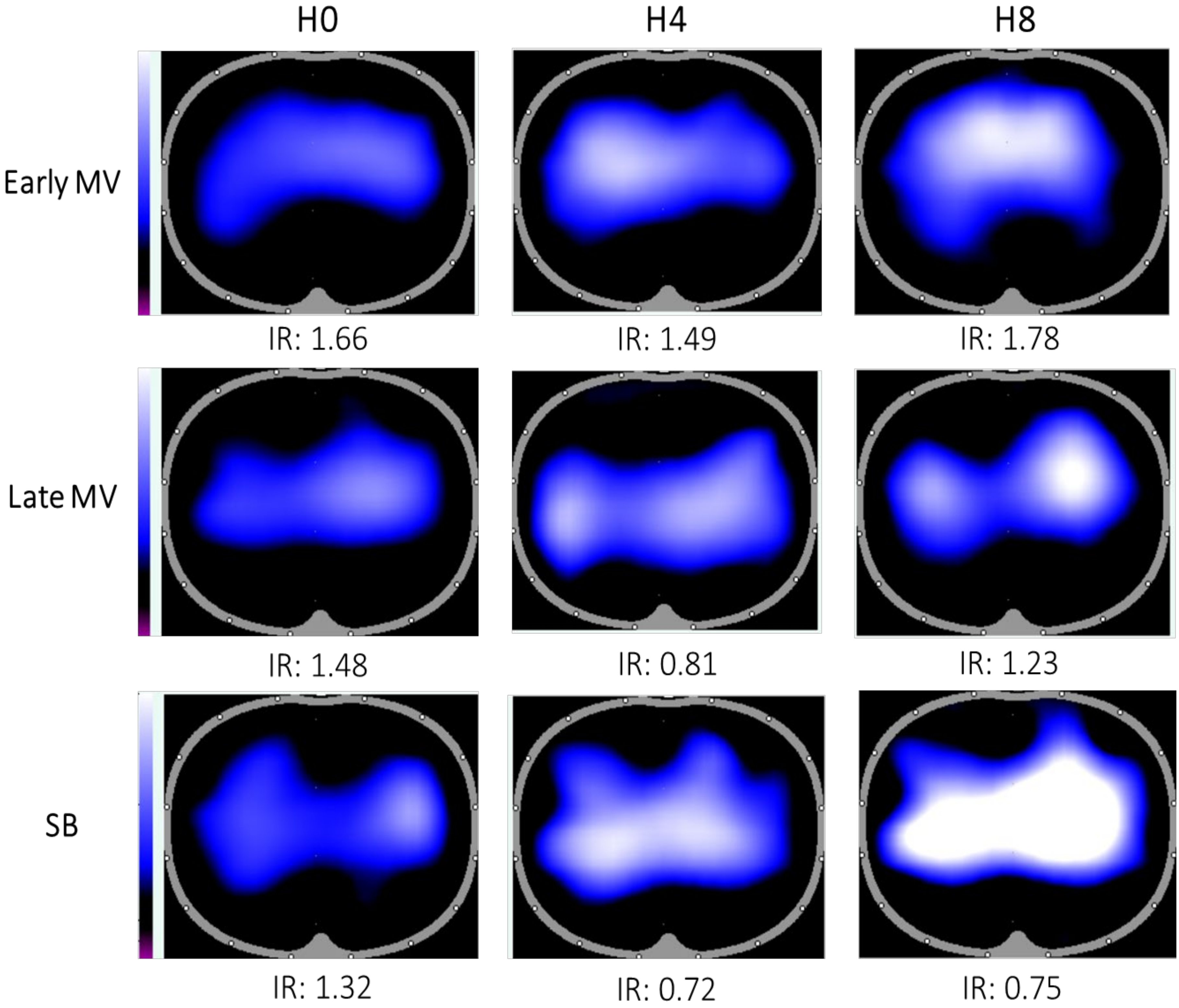

Supplementary Figure 1. Representative functional EIT figures from the three groups (Early MV, Late MV, and SB group) during the three times of the study (H0, H4, and H8). IR corresponds to Impedance Ratio.
