# Supplementary material for: Spontaneous breathing promotes lung injury in an experimental model of alveolar collapse
Source: Sci Rep. 2022 Jul 25;12:12648. doi: 10.1038/s41598-022-16446-2 (PMC9310356; doi:10.1038/s41598-022-16446-2)
Supplement: Supplementary file 2 — Supplementary Figure 2. [file 41598_2022_16446_MOESM2_ESM.pdf]

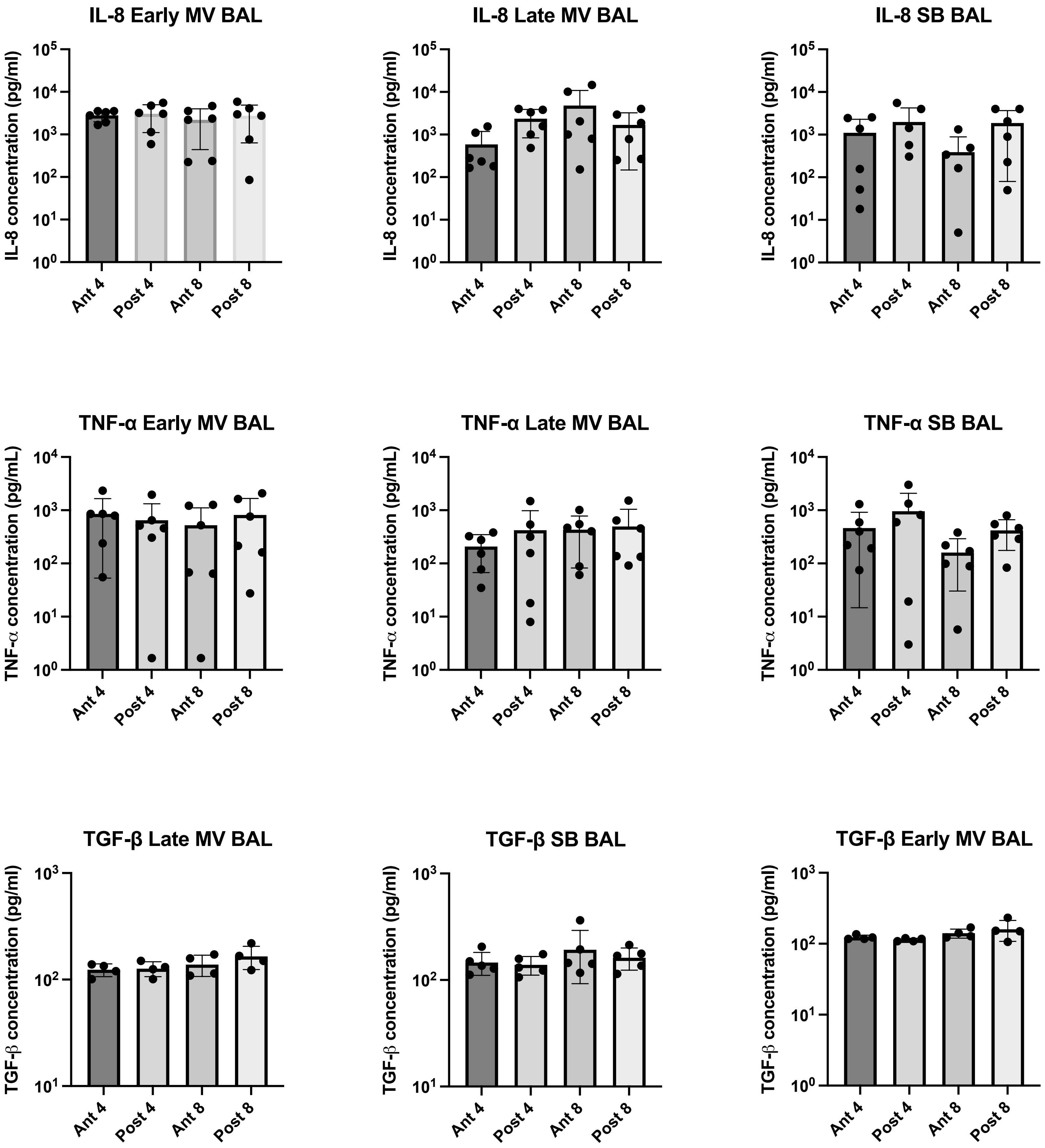

Supplementary Figure 2. Concentration of cytokines (IL-8, TNF-α, and TGF-β, pg/ml) from bronchoalveolar lavage fluid (BALF), corresponding to the Early MV, Late MV, and SB groups. Samples were obtained at H4 and H8 and from anterior (Ant) and posterior (Post) lung regions.
