# Supplementary material for: Spontaneous breathing promotes lung injury in an experimental model of alveolar collapse
Source: Sci Rep. 2022 Jul 25;12:12648. doi: 10.1038/s41598-022-16446-2 (PMC9310356; doi:10.1038/s41598-022-16446-2)
Supplement: Supplementary file 3 — Supplementary Figure 3. [file 41598_2022_16446_MOESM3_ESM.pdf]

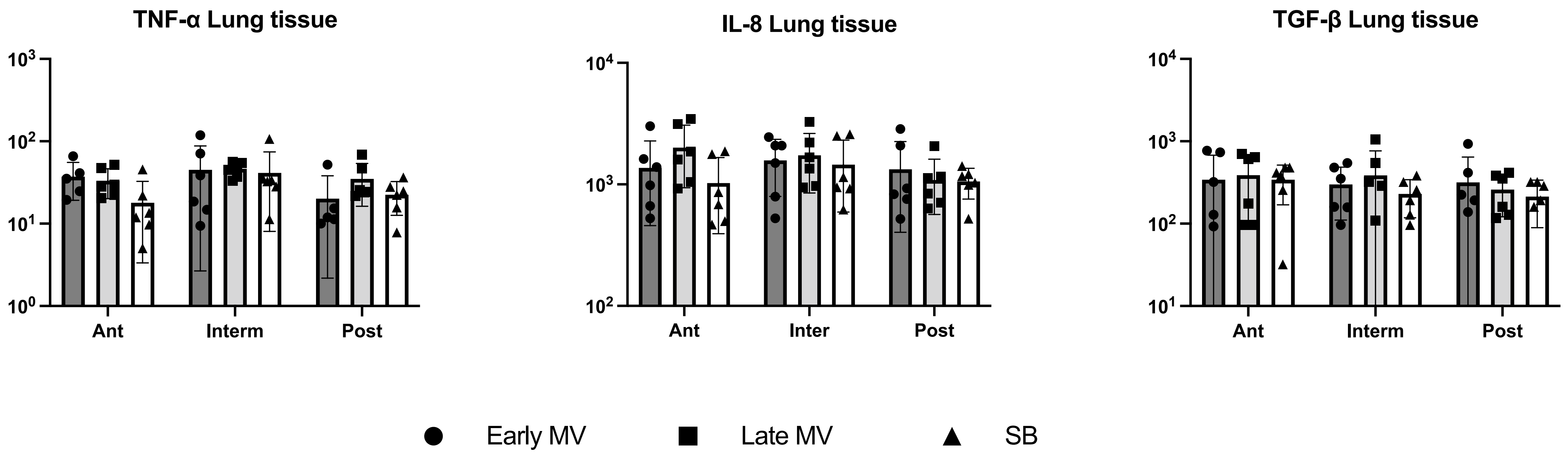


Supplementary Figure 3. Concentration of cytokines (IL-8, TNF-α, and TGF-β, pg/ml) from lung tissue homogenates, corresponding to the Early MV, Late MV, and SB groups. Samples were obtained from anterior (Ant), Intermediate (Inter), and posterior (Post) lung regions.
