# Supplementary material for: Spontaneous breathing promotes lung injury in an experimental model of alveolar collapse
Source: Sci Rep. 2022 Jul 25;12:12648. doi: 10.1038/s41598-022-16446-2 (PMC9310356; doi:10.1038/s41598-022-16446-2)
Supplement: Supplementary file 4 — Supplementary Figure 4. [file 41598_2022_16446_MOESM4_ESM.pdf]

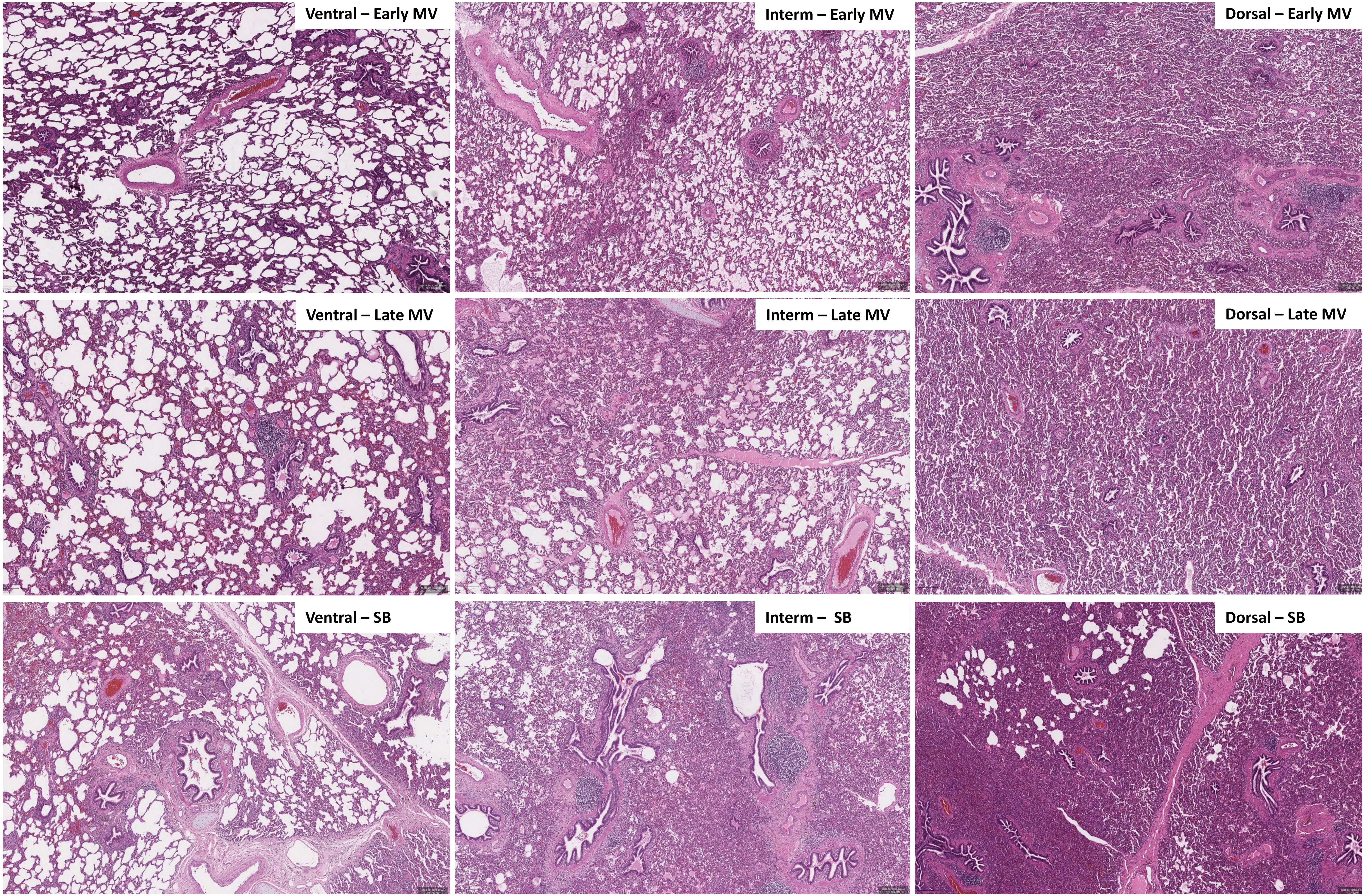

Supplementary Figure 4. Representative histology from lung parenchyma samples, corresponding to the Early MV, Late MV, and SB groups. Samples were obtained from anterior (Ant), Intermediate (Inter), and posterior (Post) lung regions.
